# The Effect of the Capping Agents of Nanoparticles on Their Redox Potential

**DOI:** 10.1021/jacs.4c02524

**Published:** 2024-07-03

**Authors:** Pavel Savchenko, Din Zelikovich, Hadassah Elgavi Sinai, Roi Baer, Daniel Mandler

**Affiliations:** ‡ Fritz Haber Research Center for Molecular Dynamics and Institute of Chemistry, The Hebrew University of Jerusalem, Jerusalem 9190401, Israel; § Institute of Chemistry, The Hebrew University of Jerusalem, Jerusalem 9190401, Israel

## Abstract

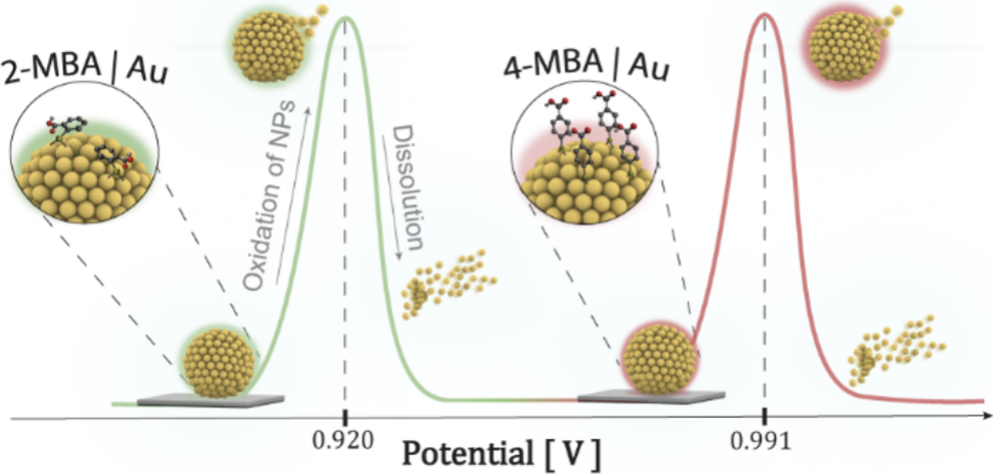

Engineered metallic nanoparticles, which are found in numerous applications, are usually stabilized by organic ligands influencing their interfacial properties. We found that the ligands affect tremendously the electrochemical peak oxidation potentials of the nanoparticles. In this work, identical gold nanoparticles were ligand-exchanged and carefully analyzed to enable a precise and highly reproducible comparison. The peak potential difference between gold nanoparticles stabilized by various ligands, such as 2- and 4-mercaptobenzoic acid, can be as high as 71 mV, which is substantial in energetic terms. A detailed study supported by density functional theory (DFT) calculations aimed to determine the source of this interesting effect. The DFT simulations of the ligand adsorption modes on Au surfaces were used to calculate the redox potentials through the thermodynamic cycle method. The DFT results of the peak potential shift were in good agreement with the experimental results for a few ligands, but showed some discrepancy, which was attributed to kinetic effects. The kinetic rate constant of the oxidation of Au nanoparticles stabilized by 4-mercaptobenzoic acid was found to be twice as large as that of the Au nanoparticles stabilized by citrate, as calculated from Laviron’s theory and the Tafel equation. Finally, these findings could be applied to some novel applications such as determining the distribution of nanoparticle population in a dispersion as well as monitoring the ligand exchange between nanoparticles.

## 1 Introduction

The realm of metallic nanoparticles (NPs) has significantly expanded over the past decades, exerting influence on many scientific disciplines such as medicine, molecular biology, and material science.^1−3^ Due to their unique electronic, chemical, and optical characteristics,^4,5^ the investigation of NPs remains an ongoing endeavor.

To understand how to tune these metallic NPs for various uses, it is essential to understand their architecture. Engineered metallic NPs are usually composed of inorganic cores that are enveloped by capping agents, i.e., functional organic ligand shells. These organic ligands control both the interface between the ligands and the core as well as between the ligands and the environment. The head groups which bind to the metal core of the NPs are critical for their stabilization and affect their size and morphology.^6−8^ In contrast, the tail groups of the ligand are responsible for NP functionality and are crucial for creating an effective repulsive mechanism between the dispersed NPs based on electrostatic and steric repulsion. In catalysis, for instance, the surface ligands can be tuned to improve the catalytic activity and selectivity of nanocatalysts.^9^ The surface functionality was also shown to affect the penetration of NPs through cell membranes.^10^

Studying the effect of the organic ligands on the properties of NPs requires the preparation of a series of metallic NPs having identical core sizes with different capping agents. The best approach for synthesizing such a series comprises ligand exchange which involves the replacement of the capping agent by other molecules dissolved in the dispersion.^11^ For example, gold NPs (AuNPs) can be controllably synthesized and stabilized with citrate following the Turkevich approach^12^ and subsequently, ligand-exchanged with alkanethiols.^13^

The organic ligands introduce several intricacies to the inorganic–organic system of NP surfaces, including steric repulsion between ligands and reactants, electron transfer, and solubility effects. These nuances are challenging to study due to the systems’ molecular scale and nanometric nature. Optical methods such as UV/vis, Fourier transform infrared (FTIR) spectroscopy, and Raman spectroscopy are widely used to characterize the NPs and the ligands.^14^ For example, by varying the organic surface ligands of ZnS and CdZnS quantum dots^15^ and silver NPs,^16^ the UV/vis absorbance signal shifts, and for some cases, the signal shape changes. Electrochemical characterization is also a powerful tool for the detection of single NPs^17^ and for studying electron transfer involving NPs.^18^ A common approach is through their oxidation employing either chronoamperometry or linear sweep voltammetry (LSV). The latter has been used to analyze the concentration, size, aggregation, and chemical properties of mostly Au and Ag NPs.^19^

Substantial work has focused on the size dependence of Au and Ag NPs on their oxidation peak potential (*E*_p_) via electrochemical dissolution. Two hypotheses have been suggested to account for the relationship between *E*_p_ and the NP size. Plieth, Henglein, and Zamborini proposed that the shift of *E*_p_ can be attributed to a modification in the redox potential (*E*^0^) of the M^0^/M^*n*+^ redox couple, for NP diameter below 40 nm.^20−23^ Accordingly, Plieth predicted a reciprocal negative shift in *E*_p_ with the radius, attributing it to a change in the Gibbs free energy associated with an increase in the surface area.^22^ Henglein explained a similar trend by calculating the equilibrium potentials from sublimation energies.^23^ Finally, Zamborini et al. showed in their pioneering experimental work that the *E*_p_ of metallic NPs was indeed proportional to the NP size according to Plieth’s theory and Henglein’s calculations.^20,21,24^ On the other hand, Compton and co-workers provided an explanation for the dependence of *E*_p_ on NP size, based on the size-dependent diffusion profiles of metal ions originating from the oxidizing array of NPs.^25,26^

In this work, we report on the effect of the capping agent functionalities on *E*_p_. Specifically, we examined the electrochemical oxidation of AuNPs stabilized by five different capping agents, i.e., citric acid, ortho and para isomers of mercaptobenzoic acid (MBA), mercaptoacetic acid (MAA), and mercaptopropionic acid (MPA). The AuNPs were adsorbed on an indium tin oxide (ITO) electrode modified by a positively charged polymer [poly(ethylenimine), PEI] and their *E*_p_ was carefully studied and calculated by density functional theory (DFT). We found that the capping agents strongly affect *E*_p_, which to the best of our knowledge has never been reported. For example, while AuNPs stabilized by MPA and MAA have an *E*_p_ that is 20 mV more negative than citrate-based AuNPs, 4-MBA stabilized AuNPs have an *E*_p_ that is 30 mV more positive than citrate-stabilized AuNPs. Furthermore, we have shown that the organic ligands have a significant effect both thermodynamically and kinetically on the electrochemical oxidation of the NPs. These findings were compared with DFT calculations, carried out for citrate, 2-MBA, and 4-MBA ligand systems. The oxidation process of the metal with and without the ligands was simulated by employing the thermodynamic cycle method on a slab model that approximately resembles AuNPs of the studied size. This is the first study, which we are aware of, where the effect of surface-adsorbed species on the metal’s redox potential was calculated. Our DFT calculations are in agreement with the experimental results implying that the small, yet significant shifts in the *E*_p_ can be associated with the metal–ligand interfacial properties of the NPs.

## 2 Results and Discussion

**Figure 1:**
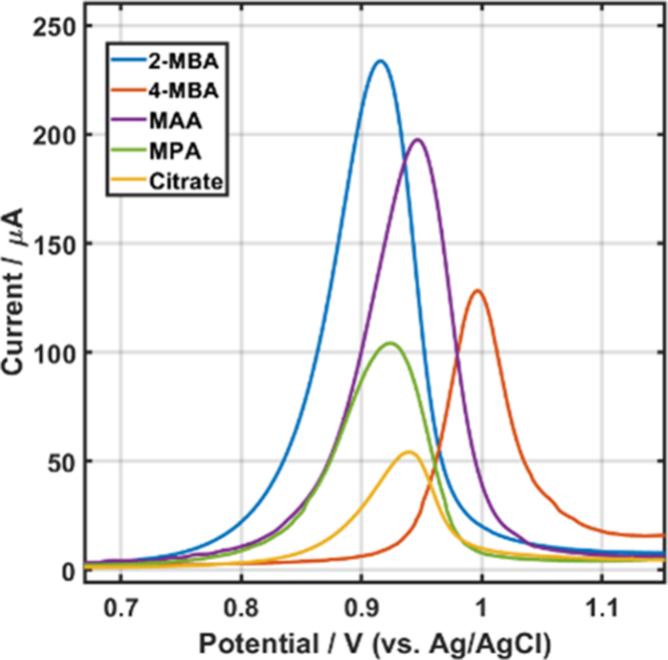
LSV of ITO/PEI electrodes recorded in 0.1 M HCl solution after adsorption of different AuNPs. The scan rate is 0.1 V s^–1^.

The electrochemical oxidation of metallic nanoparticles (NPs), such as gold and silver, adsorbed on an electrode surface usually produces a sharp Gaussian wave, whose charge is proportional to the number of adsorbed NPs. The potential of the oxidation peak, *E*_p_, is considered a thermodynamic measure indicating the ability of the NPs to be oxidized and electrochemically dissolve into the solution. We were fascinated by the distinct and significant effect of the ligand-stabilizing gold NPs (AuNPs) on the oxidation peak potential (Figure 1) observed by linear sweep voltammetry (LSV). Identical gold core NPs were stabilized by five different ligands, including citric acid, the ortho and para isomers of mercaptobenzoic acid (MBA), mercaptoacetic acid (MAA), and mercaptopropionic acid (MPA). The LSV was performed using indium tin oxide (ITO) electrodes on which a positively charged polymer, poly(ethylenimine) (PEI), was adsorbed, followed by the adsorption of the AuNPs. As can be seen, the peak potentials differ by up to 80 mV for the 2- and 4-MBA isomers stabilized AuNPs.

It is worth mentioning that numerous experiments were meticulously performed, with over 10 replicates conducted for each NP type. These extensive trials were undertaken to yield reliable statistical data and to comprehensively validate the existence of an electrochemical effect influencing the oxidation curves (Table S1).

Following this observation, we predicted that *E*_p_ is shifted merely by the difference in the chemical composition at the metal/ligand interface. In this respect, similar shifts in the oxidation waves of NPs have been reported by Zamborini and others and were attributed to differences in the size of the metallic core of the NPs. We recall that the theory proposed by Plieth^22^ correlates the standard redox potential (*E*^0^) with the NP diameter. To demonstrate that the shift of *E*_p_ in our case is not a result of the NP size but due to the metal/ligand interaction, it was essential to comprehensively characterize the NPs in the solution and on the electrode surfaces.

### 2.1 Characterization of the AuNPs

Citrate-stabilized AuNPs with a narrow size distribution were prepared following the Turkevich synthesis.^27^ This was followed by ligand exchange using thiol-based carboxylic acids, i.e., 2- and 4-MBA, MAA, and MPA. In our previous work, we demonstrated through various techniques, such as Surface-enhanced Raman spectroscopy and Fourier transform infrared spectroscopy, that the synthesis involving ligand exchange leads to a thorough replacement of the ligands.^28,29^ The challenge involved the preservation of both the uniformity and the narrow size distribution of the AuNPs (see the Experimental Section). The AuNP dispersions were carefully analyzed by DLS, UV–vis, ζ-potential, and transmission electron microscopy (TEM) after ligand exchange. The size distribution of the various NPs measured by DLS is shown in Table S1. Replacing the stabilizing ligands did not affect the size of the AuNPs, which was retained at ca. 10 nm. The assessment of AuNP stability involved the determination of the ζ-potential, as detailed in Table S1. It is anticipated due to the carboxylic acids, that the ζ-potential maintains negative at pH > 5 for all systems.^28^ All measured ζ-potentials were negative and aligned favorably with previous reports.^29^ The UV–vis spectra of the various AuNPs are shown in Figure S1.^30^ By the Mie-Gans model, spherical gold clusters with 10 nm diameter should exhibit a surface plasmon resonance at approximately 520 nm.^31^ Evidently, the singular absorbance peak at ca. 520 nm and the absence of a peak at 650 nm for all AuNPs point to negligible aggregation.^32,33^ Additionally, Figure S2 shows the high-resolution TEM images of the AuNPs on a carbon grid. The AuNPs in Figure S2 have a narrow size distribution (10.0 ± 0.5 nm measured for all of the different AuNPs using TEM imaging) with comparable morphologies that resemble the expected octahedral shape. Hence, the TEM images, the UV–vis analysis, and the DLS results confirm that the size of the synthesized AuNPs did not change by the ligand exchange process and correspond to 10 nm AuNPs.

**Figure 2:**
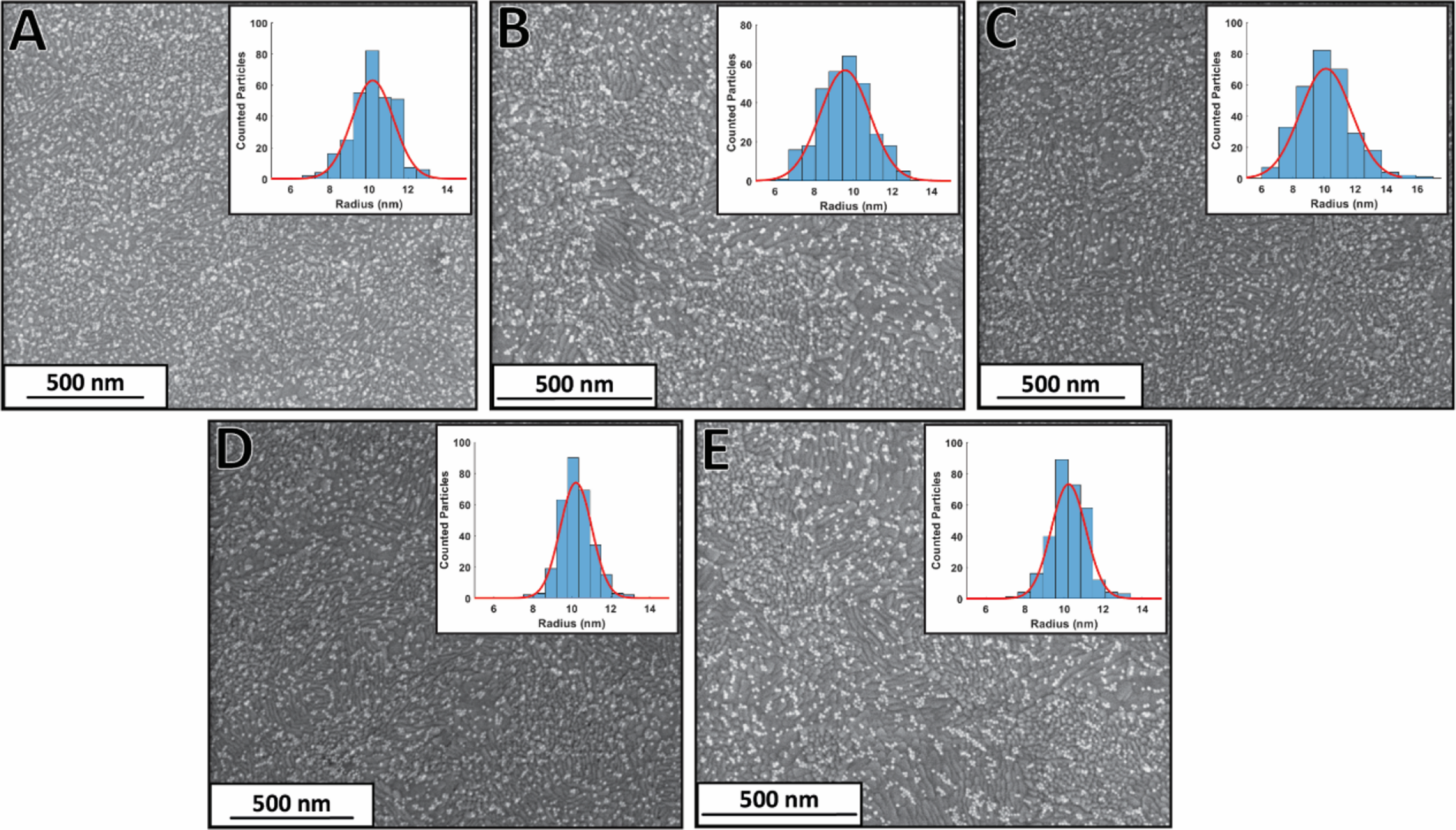
SEM images of AuNPs stabilized by (A) cit, (B) 2-MBA, (C) 4-MBA, (D) MPA, and (E) MAA. The size distribution bar plot of all AuNP types is shown in the inset for each corresponding image. For all cases, 300 particles were counted and measured.

An additional factor that impacts the redox potential is the density of the AuNPs on the surface as has already been shown.^26^ As all of these systems have identical particle size distributions, the surface density of the NPs must remain comparable, which implies that the adsorption of the AuNPs must be very well controlled. Figure 2 shows the scanning electron microscopy (SEM) images and size distribution of the ITO/PEI surfaces after the adsorption of the AuNPs with the different capping agents. Estimating the AuNPs distribution reveals that the surface density is similar for all systems (with an average of 4.94 ± 1.33 × 10^10^ AuNPs cm^–2^ as calculated from the SEM images in Figure 2), the size distribution of the NPs does not change upon adsorption, and no significant aggregation is observed. This is in accordance with our previous work, which thoroughly studied the thermodynamics of AuNPs adsorption.^29,34^ Hence, the conditions are established to form highly similar surface density arrays of AuNP bearing different ligands.

### 2.2 Electrochemical Study

The current–potential plot, i.e., the LSV, provides both the thermodynamics (for a reversible system) as well as the charge of the oxidation of the AuNPs, from which the number of the NPs on the surface can be calculated.^35^ The calculation of particles per area is described in the Supporting Information and shows that, as expected from the SEM images, the surface density of the AuNPs is similar for all of the systems and averages at 3.96 ± 2.60 × 10^10^ AuNPs cm^–2^ (Table S2).

The oxidation of gold involves the dissolution by anions, e.g., Cl^–^ and CN^–^, and therefore, the redox potential is strongly affected by the electrolyte containing such anions. For example, the standard redox potential of Au to form AuCl_4_^–^ is 0.146 V more positive than that of AuBr_4_^–^. The mechanism of the electrochemical oxidation of AuNPs that are stabilized by different ligands is not fully understood. Thus, the effect of anions such as chloride and bromide that need to reach the Au surface through the stabilizing shell, on the redox potential of AuNPs might shed light on the oxidation mechanism. Figure S3 shows that similar shifts in the redox potential of the AuNPs stabilized by the different ligands carried out in chloride and bromide electrolytes, are evident. More specifically, for all of the different ligands a shift of ca. 200 mV to more negative potential can be seen when performing the LSV in KBr instead of KCl. Furthermore, the magnitude of the shift depends on the specific ligand and follows the order: 2-MBA > Citrate >4-MBA.

**Figure 3:**
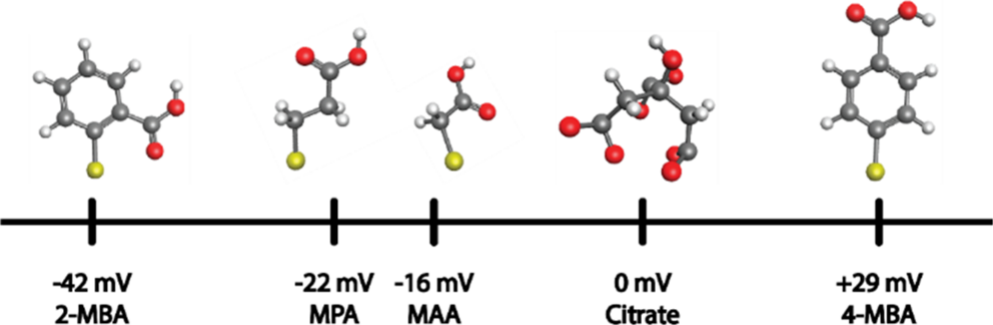
Collective data of the oxidation peak potentials for the different ligands stabilizing the AuNPs.

We recall that the redox potential of spherical AuNPs can be affected by several parameters such as the core size, electrolyte, surface density, and stabilizing ligands. Hence, to study the effect of an individual parameter, it is crucial to maintain the others constant. Therefore, we employed exactly the same core size spherical AuNPs, using the same electrolyte and adsorbing conditions in order to focus on the effect of the stabilizing ligands. Furthermore, the effect of the surface chemistry of the AuNPs on the oxidation peak potential has only been scarcely studied. Recalling Figure 1 and Table S1 clearly shows that thiol-based stabilizing ligands have a pronounced shift as compared with the parent AuNPs stabilized with citrate (Figure 3). Interestingly, whereas the peak potentials of the ω-mercapoalkanoic acids, i.e., MPA and MAA, are shifted to lower potentials as compared with the citrate AuNPs and show almost the same peak potential, the isomers of MBA behave differently. While 2-MBA is shifted to negative potentials, 4-MBA is shifted to more positive potentials. Hence, the potential difference between 2-MBA and 4-MBA is 71 mV, while that of the MAA and MPA is only 6 mV. It should be noted that the experiments were very carefully conducted which resulted in very high reproducibility.

**Figure 4:**
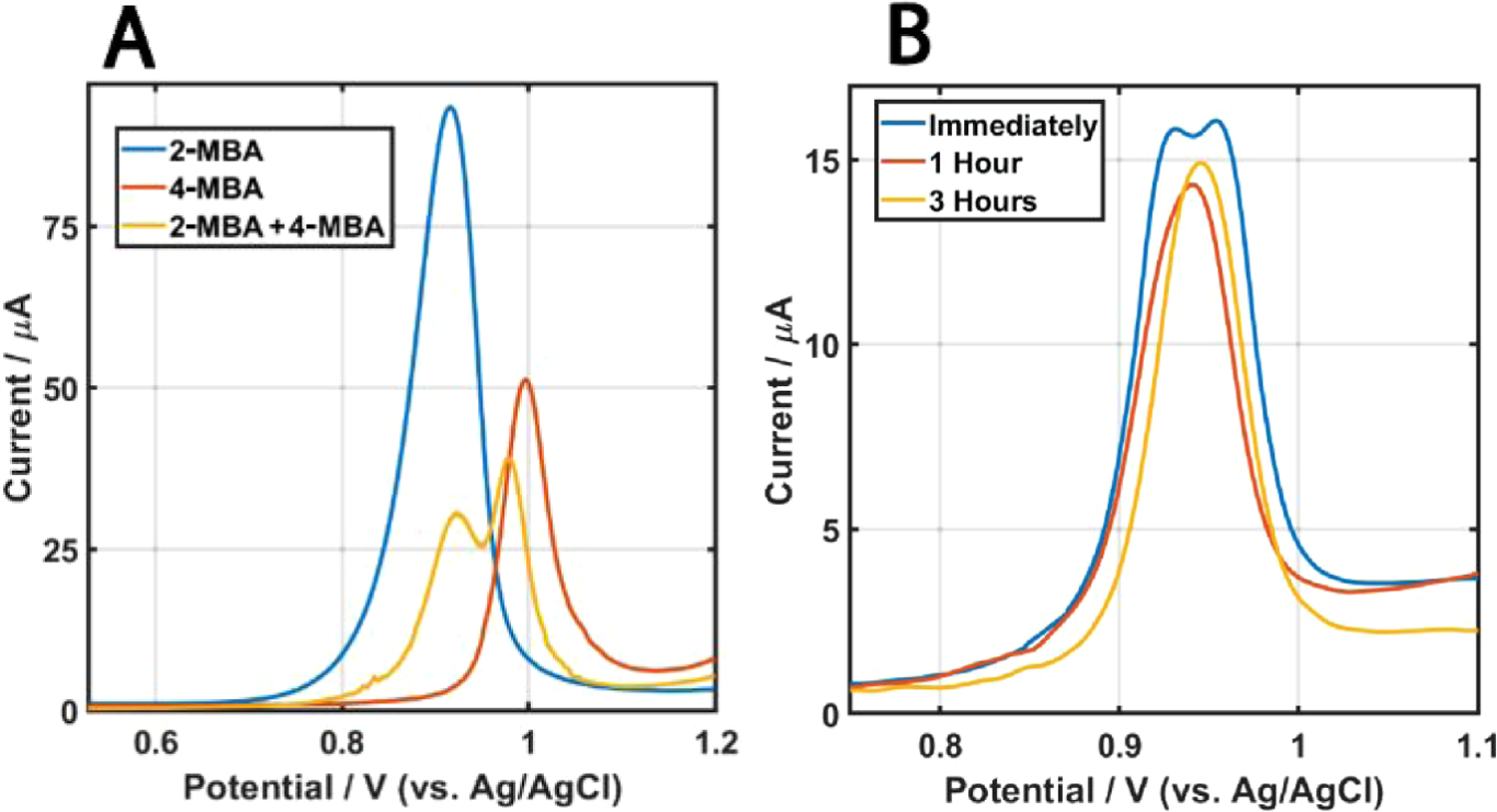
(A, B) LSV curves of ITO/PEI electrodes adsorbed with AuNPs stabilized by 2-MBA (blue), 4-MBA (red), and a 1:1 weight mixture of the 2- and 4-MBA (orange) in 0.1 M HCl and a scan rate of 0.1 V s^–1^. (B) Same as (A) but in a 1:1 weight mixture of AuNPs/cit and AuNPs/4-MBA that was stirred for different times.

One of the possible applications of the shift in the peak potential is the ability to differentiate between AuNP populations in solution. Accordingly, we studied the LSV of AuNPs adsorbed from a mixture containing a 1:1 (wt) ratio of 2- and 4-MBA. Figure 4A shows that the oxidation curve of the mixture (yellow curve) has two distinct peaks that perfectly fit the individual peaks of each of the respective particles (red and blue curves). Furthermore, Figure 4B demonstrates that this approach can also be applied to monitor the exchange of ligands. Specifically, the LSV of a 1:1 weight mixture of AuNPs stabilized by citrate and 4-MBA is shown immediately upon mixing and after 1 and 3 h. It can be seen that the LSV changes as a function of time implying ligand exchange between the 4-MBA to the citrate AuNPs. This causes the peak potentials to shift and converge until the appearance of only one peak at close to the oxidation potential of 4-MBA or the mixture of cit and 4-MBA capping agents.

### 2.3 Theoretical Prediction of the Standard Redox Potential

**Figure 5:**
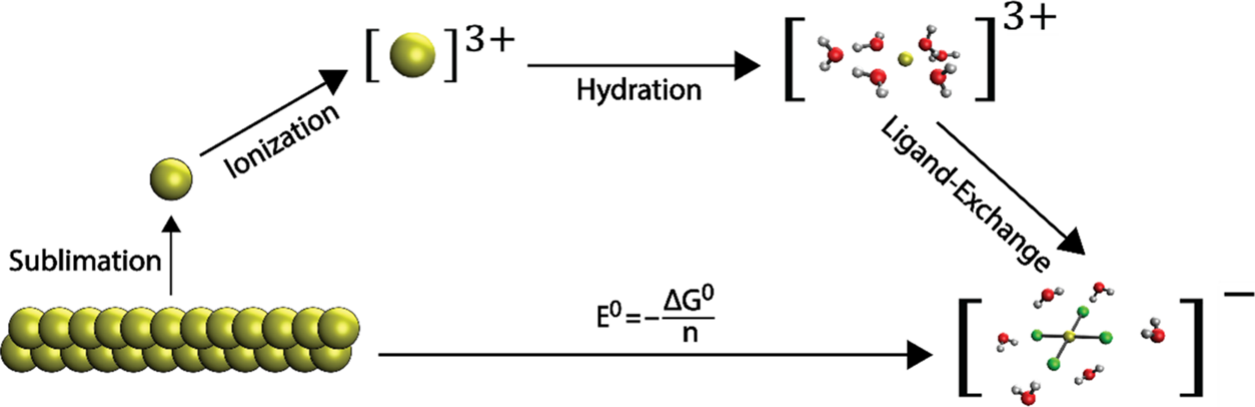
Scheme of the thermodynamic cycle used for calculating *E*^0^ illustrated for the reaction in eq 1. See text for a description of the stages of calculation.

To estimate the standard redox potential using DFT, a thermodynamic cycle is commonly used (Figure 5).^36−39^ In the case of the AuNPs, the oxidation happens through the reaction:^40,41^
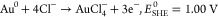
1where *E*_SHE_^0^ is the standard redox potential vs the standard hydrogen electrode (SHE), which is expressed by using the Gibbs free energy change, Δ*G*^0^. *E*^0^ is related to Δ*G*^0^ through eq 2, where *n* is the number of electrons involved in the oxidation of one Au atom.
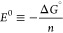
2

The SHE potential is related to *E*^0^ by the absolute potential value of the hydrogen electrode^42^ and is expressed as
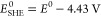
3

The free energy change of the process (Figure 5) is expressed through the sublimation energy, Δ*E*_sub_ (where the metal atom is removed from the surface of the NP); the gas-phase atomic ionization energy, Δ*E*_ion_ (of *n* electrons); the hydration energy of the ion in water, Δ*E*_hyd_ (creating an intermediate hydrated ion); and finally, the ligand exchange energy, Δ*E*_le_ (in our case with 4 Cl^–^ in the solution) of water with other molecules or ions to create the final complex.

Each step is then calculated separately, and *E*^0^ is expressed through the combination of all of the energies:
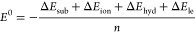
4

Although this approach is widely used to calculate redox potentials of species in solution, with moderate success, the oxidative dissolution of metals remains a challenge. Specifically, the sublimation energy is treated as a cohesive energy of the bulk material, and only experimental results were considered for it. In this work, the whole oxidation process of the metal surface was considered theoretically and was qualitatively compared to experimental data.

### 2.4 Analysis of the Sublimation Energy

The first step of metal oxidation is the removal of metal atoms from the slab into the gas phase. Usually, this is referred to as cohesive energy, defined as the energy required to remove a neutral atom in the electronic ground state from a bulk material at 0 K and 1 atm. However, in this case, a more appropriate term would be the sublimation energy, corresponding to the transition from the solid to the gas phase. As the electrochemical oxidation reaction occurs at the interface between the electrode surface and the electrolyte solution, the atoms are initially extracted from the top layers of the surface rather than the bulk. Moreover, sublimation energy is expected to be affected by different electrode-surface-adsorbed ligands since that can change the electronic structure of the surface.

We evaluated sublimation energies by the following procedure: First, we established the slab’s geometry (together with the adsorbed species, if present) by geometry optimization, determining its relaxed energy *E*_slab_. Next, we identified the atom with the weakest bond to the surface: each surface atom was removed relaxing the system and searching for the highest relaxed surface-hole energy *E*_slab_^′^. We checked an alternative method, which gave almost identical results for *E*_slab_^′^, where we lifted an atom to a height *H* above the surface and relaxed the remaining atoms in the slab (Figure S4 shows the convergence to a limit of the sublimation energy as a function of *H*). The sublimation energy is defined as
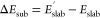
5

We also checked the removal of atoms from subsurface layers, (essentially, determining the subsurface stability after desorption, see Figure S5). We concluded that atoms indeed dissolve from the uppermost layer during metal dissolution.

The transition from NPs to surfaces raises an important point of discussion regarding the calculations. The AuNPs used in the experimental part are relatively large, 10 nm in diameter, containing O(10^5^) atoms. It is not feasible to treat such a large system by DFT, and therefore, surfaces with periodic boundary conditions were used instead. In many aspects, AuNPs of this size have near-bulk behavior; for example, in catalytic processes, the turnover frequencies demonstrate trends consistent with bulk when considering NPs with a diameter of 10 nm.^43,44^

In electrochemistry, following the theory proposed by Plieth,^22^ the standard redox potential of spherical metallic NPs undergoes a shift relative to the redox potential of the bulk metal. This shift is proportional to the inverse of the NP radius and is a consequence of the change in the surface free energy between the bulk surface and dispersed NPs with an equivalent number of atoms. For a 10 nm particle, the shift from bulk is 19 mV to more negative potentials, a very subtle shift compared to 4 nm particles and smaller, where the potential shifts are significantly larger (50 mV, see Figure S6).

In 10 nm AuNPs, the surface is composed of intersecting Au(111) facets due to the prevalence of the decahedron and truncated octahedron morphologies.^26^ Because these structures have flat surfaces, unlike spherical NPs, we can overlook the curvature of the AuNPs except near the edges where these flat surfaces intersect.

To conclude, considering the NPs size (employed in this work) relative to the atomic scale involved in sublimation, we can approximate that an atom removed from the NP behaves similarly to one removed from the bulk surface. Thus, we can concentrate on the differences in the NP’s chemical surface composition and its effect on the redox potential.

### 2.5 Characterization of the Ligand Adsorption Modes

The Au(111) surface was chosen as the primary surface for the sublimation studies, as the AuNPs synthesized with citrate mainly consist of Au(111) facets. Citrate is used to control the growth during the synthesis, and as it has the strongest interaction with the Au(111) surface, it directs the growth of the Au seeds by forming these facets.^45^

**Figure 6:**
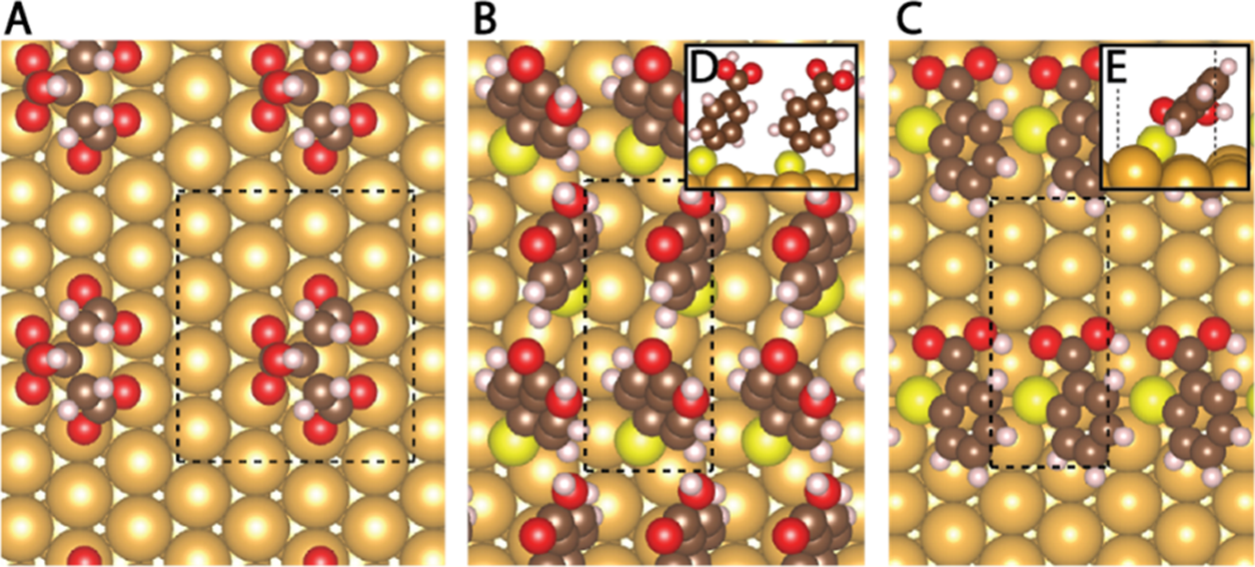
Adsorption modes of (A) citrate in a 2√3 × 4 unit cell, (B) 4-MBA, and (C) 2-MBA in a √3 × 4 unit cell. (D) Side view of two 4-MBA molecules adsorbed on an hexagonal closest packed (HCP) and face-centered cubic (FCC) sites with angles of α = 65 and 80° to the surface, respectively. (E) Side view of a 2-MBA molecule adsorbed on a Bridge-FCC with an angle of α = 45° to the surface.

The adsorption modes of the 4-MBA and citrate on Au(111) were studied intensively and are well established from STM imaging and reductive desorption (RD) studies. For the citrate at *pH* ≈ 7, all three carboxylic groups are expected to deprotonate and coordinate with the surface (*pK*_*a*_ values are 3.128, 4.761, and 6.396, respectively^46^). Therefore, all oxygen atoms in the carboxylic groups bind on the quasi-top site of the Au(111) lattice, creating a 2√3 × 4 unit cell with a single citrate anion as shown in Figure 6A.^45,47^

The 4-MBA molecules are well-known to adsorb on the Au(111) surface as a self-assembled monolayer in a √3 × 4 unit cell with a surface coverage of θ = 0.25 which consists of two 4-MBA molecules per unit cell.^48−50^ The two 4-MBA molecules have different adsorption sites in the unit cell, i.e., one is adsorbed at the HCP site, while the other is at the FCC site, as seen in Figure 6B.

In contrast to the other ligands, the adsorption modes for the 2-MBA molecule are less studied. However, it was shown using Raman spectroscopy, that a bond is formed between the oxygen on the carboxylic group (of the 2-MBA) and the gold surface during adsorption, thereby creating a second surface-ligand interaction, in addition to that of the thiol group.^29^ Furthermore, STM imaging and RD studies showed that 2-MBA molecules arranged in ordered domains of a √3 × 4 lattice with a lower surface coverage of θ = 0.125^51^ compared to 4-MBA, meaning that there is a single molecule in the unit cell. Yet, the organization of the 2-MBA inside this unit cell is unclear.

The search for the most stable configuration of the 2-MBA was conducted by relaxing many permutations of the molecule placed in a √3 × 4 unit cell. The configurations were created by randomly rotating the molecule along all three axes by varying angles (with consideration to geometric constraints) in each one of the adsorption sites (FCC, HCP, bridge, bridge-FCC, bridge-HCP). The bridge-FCC and bridge-HCP sites denote the displacement of the bonding atom from the bridge site toward the FCC and HCP sites, respectively. Each configuration undergoes geometry optimization through the relaxation of the adsorbed molecule and the three top Au layers. We examined several hundreds of configurations, comparing the binding energies of the various adsorption sites. In conclusion, we found that the most favorable adsorption of 2-MBA is the bridge-FCC site in a √3 × 4 unit cell with the oxygens of the carboxylic group rotated parallel to the Au surface (Figure 6C).

### 2.6 Evaluation of the Redox Potential Shifts

First, we compare the calculated redox potential of a clean Au slab, without any adsorbates, to the experimental potential of an Au electrode. The potentials are vs SHE. The results are shown in Table 1.

**Table 1:** Estimate of the Standard Redox Potential of a Clean Au Surface^a^^37^

Δ*E*_sub_ (eV)	ionization energy (eV)	hydration energy (eV)	ligand exchange energy (eV)	standard redox potential (V)	Δ from experiment (mV)
4.43	66.24	–51.45	–2.92	1.003	3

a All energies are calculated according to the computational methods described in the Experimental Section. Δ is the deviation from the standard redox potential, which is 1.00 V.^37^

It is evident that the computed standard redox potential is nearly identical to the experimental data. However, the AuNPs in our experiments are coated with a ligand, e.g., citrate, therefore, a naked AuNP system cannot be used as a valid reference system. The redox potential of the Au slabs with the different ligands is given vs citrate in Table 2.

**Table 2:** Calculated Binding Energy *E*_b_, Sublimation Energy Δ*E*_sub_, and Standard Redox Potential of All Studied Systems^a^

	*E*_b_ (eV)	Δ*E*_sub_ (eV)	standard redox potential (V)	potential Δ from citrate (mV)
clean Au		4.43	1.003	
citrate	–5.33	3.90	0.826	
2-MBA	–3.01	3.86	0.811	–15
4-MBA	–2.27	3.70	0.760	–66

a The potential shifts of the 2- and 4-MBA systems vs. that of the citrate are shown in the right column.

The binding energy difference between the 2- and 4-MBA systems arises from the difference in the binding of the ligand to the electrode surface. The 4-MBA molecules organize as a self-assembled monolayer bound to the surface solely through the sulfur, whereas the 2-MBA has an additional bond through the O atom of the carboxylic group. This is termed the anchoring effect, where the oxygens from the carboxylic group approach the Au surface, creating an Au–O bond as discussed in the adsorption section. This anchoring effect increases the binding energy (*E*_b_ in Table 2) resulting in a stronger adsorption to the surface.

The difference in the sublimation energies between both Au-MBA systems arises from the adjustment of the metallic layer to the adsorption of each MBA isomer. Interestingly, our calculations imply that the Au atom that primarily detaches during sublimation in both Au-MBA systems is one of the atoms that participate in the Au–S bond, that binds the adsorbed MBA to the surface. More specifically, the sulfur is pulling the Au atoms, contracting the Au–S bond (to approximately 2.40 Å), and as a result weakening the Au–Au bonds of the surface uppermost layer. Consequently, these Au atoms are elevated above the average surface height during the relaxation of the slab (0.51 Å for the 2-MBA and up to 0.60 Å for the 4-MBA molecule, see Figure S7) which makes them more susceptible to dissolution. This phenomenon is also evident in small clusters, where the 2- and 4-MBA molecules cause the Au–Au bonds to contract within clusters consisting of just a few atoms (depicted in Figure S8). By examining these small systems, the sublimation energies of the extreme case of a few Au atoms can be compared to the sublimation from flat slabs. The AuNPs decrease in size during the electro-dissolution process until they are fully dissolved. However, as we cannot explain the entire dissolution mechanism using DFT, examining these small systems provides insight into the behavior and energetics when only a few atoms remain on the surface. The sublimation energies of the small clusters are much lower than those of the Au slabs coated with ligands (up to a difference of 2.44 eV for a flat slab adsorbed with the 2-MBA molecule and an Au4 cluster with the same molecule, as seen in Figure S8). Thus, by examining the most extreme cases, it can be concluded that the threshold energies required to initiate the oxidation process are determined by the initial Au atom cleavage from the slab.

The calculation results in a 0.51 eV difference between the sublimation energies of the citrate and the clean Au, which has a pronounced effect on the standard redox potential of the system. Although the clean Au yields reliable results, the redox potential of the citrate system deviates by 177 mV from the clean Au. Based on these calculations, it is evident that all of the ligands reduce the standard redox potential of the Au slab substantially. This potential shift is attributed to the Au layer elevation, specifically the Au atom bound to the ligands, resulting in less energetic sublimation.

When comparing the results between the citrate reference and the MBA systems, both the 2- and 4-MBA ligands further reduce the sublimation energy of the Au. The redox potential of Au in the 2-MBA system is shifted by −15 mV relative to the citrate. This trend is consistent with the negative shift of the 2-MBA in the experimental system, where the reported potential shift is −42 mV. However, in contrast to the experimental findings, the calculations suggest a potential shift of −66 mV of the 4-MBA as compared to the citrate, whereas a positive shift of ∼30 mV is observed experimentally. Both computed results suggest a less energetic dissolution of the Au surface (as compared to the citrate) when the MBA isomers are adsorbed.

A plausible explanation could be attributed to the different packing of the adsorbed isomers on Au. We have shown both experimentally and computationally that 4-MBA forms a significantly more compact SAM on Au as compared with 2-MBA and citrate. The high density of the adsorbed 4-MBA pulls more effectively the top Au surface layer causing the sublimation of Au atoms to be more favorable thermodynamically. Yet, the gold atom is required to cross a dense (and more hydrophobic) organic layer to fully dissolve. Since the calculation takes into account only the initial and final states of the gold, this kinetic barrier does not affect the calculated potentials, and yet, is expressed in the experimental results.

**Figure 7:**
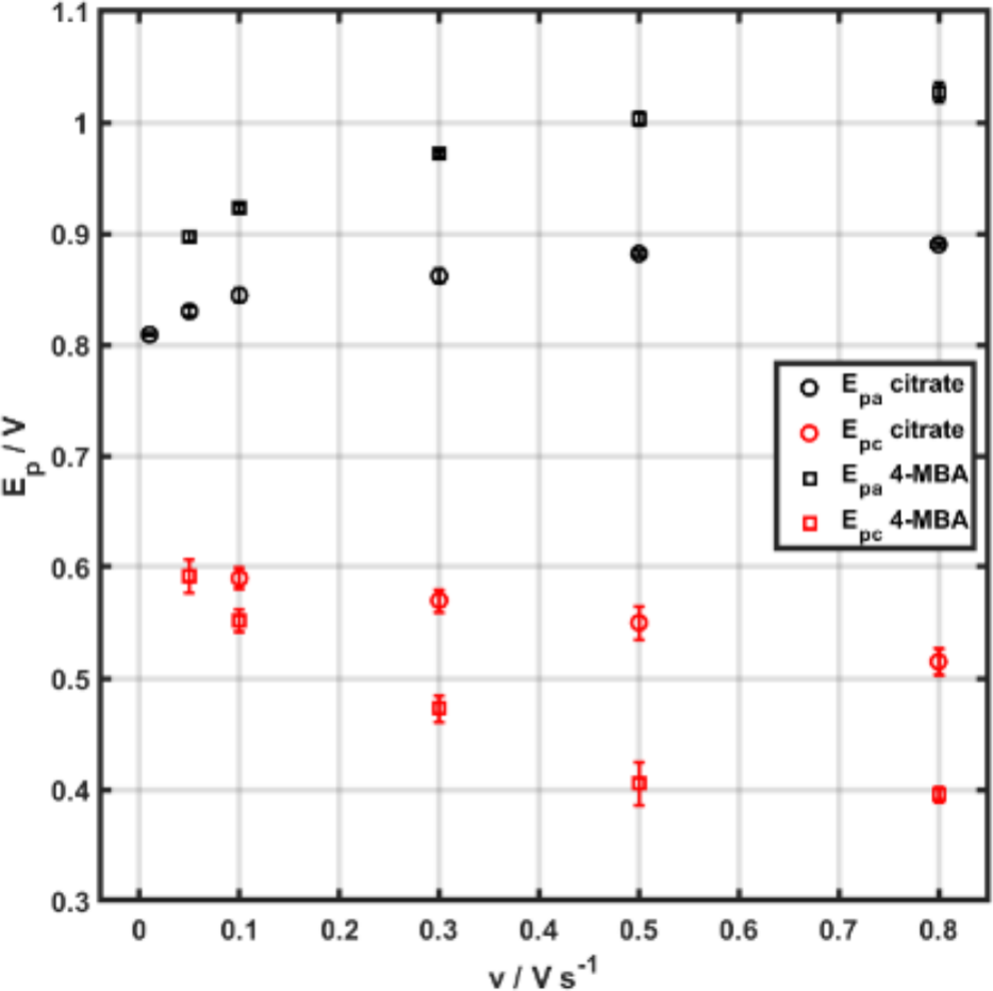
Anodic (black) and cathodic (red) peak potentials, *E*_p_, plotted against the scan rate, *v*, obtained from CV scans of AuNPs stabilized by citrate (circles) and 4-MBA (squares).

This poses an interesting question of whether the oxidation peak potential of the adsorbed AuNPs is dependent solely on the thermodynamics or is also kinetically controlled. Interestingly, the majority of studies have attributed the peak potential to a thermodynamic value.^35,52^ To the best of our knowledge, kinetic studies of NPs oxidation were performed solely for single NP oxidation using the nano impact method.^53,54^ Evidently, the kinetics of the oxidation process should be revealed by a dependence of the redox peak potential on the scan rate in cyclic voltammetry (CV). Hence, we studied the effect of the scan rate on the CV of AuNPs stabilized by citrate and 4-MBA (Figure S9). The anodic and cathodic peak potentials clearly show positive and negative potential shifts, respectively, with increasing scan rates as seen in Figure 7. It is worth mentioning that *iR* compensation was employed for all CV scans as relatively high scan rates (i.e., 0.05–0.8 V s^–1^) were applied.

It is evident that the process of capped AuNP dissolution involves multiple stages and is not straightforward due to the intricate nature of the system. To exclude the possibility of the surface being responsible for the electron transfer rate-limiting step, we conducted additional experiments on different surfaces such as Pt (Figure S10) which is a more well-defined electrode as compared with an ITO electrode. Notably, the potential shifts observed on a thoroughly cleaned Pt surface closely resemble those seen on an ITO surface. This strongly suggests that the type of electrode surface does not govern electron transfer, thus, indicating that electron transfer from the AuNPs to the electrode surface is likely not the rate-limiting step.

The oxidation of metallic NPs such as AuNPs involves multiple stages and as such we employed two approaches for studying it, i.e., the Laviron theory and the Tafel equation. According to Laviron’s theory,^55^ which was primarily applied to adsorbed molecular species that undergo an irreversible electron transfer process, the cathodic peak potential is expressed as
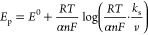
6where *k*_s_ is the standard heterogeneous rate constant; *n* is the number of electrons transferred; α is the charge transfer coefficient; *v* is the selected scan rate; and *R*, *T*, and *F* are the gas constant, temperature, and Faraday constant, respectively. Figure S11 depicts the dependence of *E*_p_ as a function of the natural logarithm of the scan rate for the different systems. It can be seen that a clear linear dependence is obtained, in good agreement with the Laviron equation for both systems. The charge transfer coefficient α calculated from the slope of the linear curves for the AuNPs stabilized by citrate and 4-MBA is α_cit_ = 0.543 and α_4-MBA_ = 0.818.

When *n*Δ*E*_p_ > 200 mV (Δ*E*_p_ is the peak potential difference between the anodic and the cathodic peaks), *k*_s_ can be calculated through the following equation:^55,56^
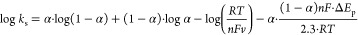
7

By inserting α into eq 7, *k*_s_ were calculated for both systems and are *k*_s_cit__ = 0.38 ± 0.10 s^–1^ and *k*_s_4-MBA__ = 0.17 ± 0.02 s^–1^ showing, as expected, independence on the scan rate. The thermodynamic standard potentials were extracted by using the kinetic parameters α and *k*_s_ from the Laviron equation (*E*_cit_^0^ = 0.972 ± 0.005 V and *E*_4-MBA_^0^ = 1.101 ± 0.002 V) and are following the previously shown experimental results for the redox shifts (Figure 3). These kinetic parameters can help explain the DFT calculations; however, the application of Laviron’s approach to such a system is questionable. Moreover, and as mentioned above, electron transfer between the metallic NP and the electrode is likely not to be the rate-limiting step. The electrochemical oxidation of AuNPs also involves ion transfer that is coupled to the electron transfer. To the best of our knowledge, Laviron’s theory has never been applied to such cases. Therefore, we have also examined the CV of the NP oxidation using the Tafel equation:
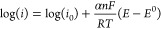
8where *i* is the current and *i*_0_ is the exchange current density and is linearly proportional to *k*_s_.

Figure S12 shows the LSV and the corresponding Tafel plots for the oxidation of AuNPs stabilized by both citrate and 4-MBA for different scan rates. For each of Tafel plots, the best linear dependent part was used for extracting the intercept, which is related to *i*_0_ and *k*_s_. *i*_0_ cannot be fully determined due to the unknown of the surface concentrations of the oxidized and reduced species. Yet, we have shown (Figure 2) that the concentration of the AuNPs on the surface is similar for both systems. At the same time, we can compare the ratio of the intercepts derived from the Tafel equation and that obtained from Laviron’s theory (Table 3). Hence, treating the kinetic data with two different and independent approaches results in very similar ratios between the kinetic parameters describing the rate constants of the two systems. This suggests that the oxidation of AuNPs stabilized by citrate and 4-MBA is indeed kinetically controlled and conceivably can be analyzed by applying both the Laviron and Tafel approaches. Moreover, unlike Laviron’s theory, which exclusively considers a single point (*E*_p_) on the voltammogram, the Tafel theory requires the fitting of a segment of the voltammogram.

**Table 3:** Ratio between the Intercepts Obtained from the Tafel Equation and the Kinetics Parameters Obtained from Laviron’s Theory for the Electrochemical Oxidation of AuNPs Stabilized by Citrate and 4-MBA

Tafel	
Laviron	

As these kinetic rate constants differ between the two AuNP systems, having the same density of AuNPs on the electrode surface, it can be concluded that the ligand shell stabilizing the AuNPs affects the kinetics of the oxidation process. The reaction rate constant of the citrate-based AuNPs is larger by a factor of 2 than that of the 4-MBA-based AuNPs (as extracted from both methods), implying a faster, and more reversible oxidation reaction of the citrate system. The smaller reaction rate constant of the 4-MBA indicates slower kinetics and longer time required to reach equilibrium. These results strengthen our suggestion that Au(III) from the particles has to cross the denser ligand shell of the 4-MBA (as compared to citrate) to diffuse into the solution. This can explain the difference between the DFT calculations, which are based only on thermodynamics, and the experimental data for the AuNPs stabilized by the 4-MBA, which also includes the kinetic barriers.

## 3 Conclusions

This study has focused on an interesting and nonstudied phenomenon whereby the electrochemical oxidation peak potentials of Au nanoparticles (AuNPs) that are adsorbed on an electrode surface are strongly affected by the ligands that stabilize the nanoparticles (NPs). Therefore, we conducted a comprehensive experimental and computational analysis of the peak potentials of AuNPs stabilized by aromatic and aliphatic thiols and citric acid. The shifts of the peak potentials are highly reproducible and can be used to distinguish between adsorbed NPs through their surface chemistry. Furthermore, this simple analysis enables the detection of different NP populations in the solution.

Understanding the origin of these shifts was accomplished by DFT simulations of the ligand adsorption modes on Au surfaces, and calculating the redox potentials through the thermodynamic cycle method. The DFT results of the peak potential shift were in good agreement with the experimental results of the 2-MBA, however, could not fully predict the magnitude of the experimental shift of the 4-MBA system. This discrepancy encouraged us to study the kinetic behavior of the system which manifested as a significant factor in the redox peak potential shifts of the different AuNPs. Our findings indicate that the slower kinetics of the 4-MBA system, as opposed to citrate, suggests that the hydrophobic 4-MBA layer contributes to the observed kinetic shift of the peak potential. Yet, we have shown that the organic ligands, i.e., capping agents, which stabilize the metallic NPs, have a significant effect both thermodynamically and kinetically on the electrochemical oxidation. DFT calculations are a powerful tool for such electrochemical studies, where the thermodynamics of the system plays a major role. Dealing with kinetic effects, which we are currently exploring in our lab, will further strengthen DFT calculations in such interesting systems.

## 4 Experimental Section

### 4.1 Materials

Ethanol (reagent grade) was ordered from J.T. Baker. Acetone (AR grade) was obtained from Gadot, Israel. Hydrochloric acid (gradient grade) was obtained from Loba Chemie. Trisodium citrate (99%) was obtained from BDH. Chloroauric acid hydrate (HAuCl_4_·3H_2_O, 99.9%), potassium hexacyanoferrate (III), 2-mercaptobenzoic acid (99%), 3-mercaptobenzoic acid (99%), 3-mercaptopropionic acid (99%), thioglycolic acid (98%), 4-mercaptobenzoic acid (99%), poly(ethylenimine) (PEI) aqueous solution (0.72 mg·mL^–1^, *M*_w_ = 800 g·mol^–1^), potassium bromide, potassium chloride, and sodium hydroxide were purchased from Sigma-Aldrich. All of the chemicals were used as received. One-side-coated ITO plates (7 mm × 50 mm × 0.7 mm) were purchased from Delta Technologies (CG-601N-CUV, Stillwater, MN). Dialysis tubing membrane (MWCO 12–14 kDa) was ordered from Medicell Membranes Ltd. (Liverpool, London). Ultrapure deionized water (Easy Pure UV, Barnstead) was used for all aqueous solutions.

### 4.2 Instruments

Cyclic voltammetry (CV) and linear sweep voltammetry (LSV) were conducted with a CHI-630 (CH Instruments, Inc., Austin, TX) potentiostat using a three-electrode setup glass cell. An Ag/AgCl (KCl 1 M) was used for the aqueous solution. A Pt wire was used as the counter electrode. Extra-high-resolution scanning electron microscopy (XHR-SEM, Magellan XHR 400L, FEI) and high-resolution transmission electron microscopy (HRTEM, Tecnai G2 F20) were used to characterize the adsorbed NPs. ζ-Potential was measured by dynamic light scattering (Zetasizer, Malvern ZS). The conductivity of the dialysis bath was measured by an Exstik EC400 conductometer (EXTECH Instruments). UV measurements were performed using a Shimadzu 300pc spectrophotometer (600–450 nm).

### 4.3 Procedures

#### 4.3.1 Synthesis of AuNPs

Gold NPs (∼10 nm diameter) stabilized with citrate (AuNPs-cit) were synthesized based on the Turkevich method^57^ with some minor changes. In particular, 97 mg of trisodium citrate was dissolved in 150 mL of water (2.2 mM) and heated for boiling under vigorous stirring. Then, 1 mL of an aqueous solution of 25 mM HAuCl_4_·3H_2_O was added and stirred for 10 min until a red color was obtained.

#### 4.3.2 Ligand Exchange of AuNPs by ω-Mercaptocarboxylic Acids

The AuNPs-cit were ligand-exchanged by different thiols such as ortho and para isomers of mercaptobenzoic acid (MBA),mercaptoacetic acid (MAA) and mercaptopropionic acid (MPA). The pH of the aqueous thiol solutions was adjusted from 2 to 6 using small amounts of NaOH (0.1 M). Next, three equivalents of a 0.6 mL solution (3 × 10^–6^ mol) of thiol at pH 6 were introduced into 1 mL of the seed solution. The resulting mixture was diluted with 9 mL of water and gently agitated at a rate of 90 strokes per minute for a duration of 30 h. Notably, the solution’s color remained unchanged throughout the entire procedure.

Subsequently, these AuNP dispersions (totaling 100 mL) were transferred to a dialysis tubing membrane to remove excess ligands. The dialysis process was initiated by placing the AuNP dispersions in contact with ultrapure deionized water at room temperature, while continuously stirring for a period of 24 h. The volume of ultrapure deionized water in the dialysis chamber was consistently maintained at 2 L. To monitor the progress of dialysis, the conductivity of the ultrapure deionized water was measured at hourly intervals.

#### 4.3.3 Functionalization of ITO Electrodes with Different AuNPs

ITO substrates were cleaned by sonication in acetone, ethanol, and deionized water (twice) for 10 min and then dried under N_2_. Next, the ITO substrates were immersed in a poly(ethylenimine) (PEI) aqueous solution (0.72 mg·mL^–1^) for 2 h with mild shaking (100 strokes/min) and later were washed for 24 h by mild shaking (100 strokes/min) with deionized water. This treatment avoided the excess of PEI chains that were not completely adsorbed to the ITO. Finally, the ITO/PEI substrate was directly immersed in an aqueous solution of the negative AuNPs coated by different ligands for 1 h. Then, the plates were dried with a flow of Ar for 1 min. This treatment led to electrostatic attachment between the ITO surface and the AuNPs. For the experiment where we studied the electrochemical oxidation of AuNPs from the ITO electrode, we used electro-oxidation using a three-electrode cell in a 0.1 M HCl solution. An LSV scan from 0.2 to 1.3 at 0.1 V s^–1^ was performed to oxidize the AuNPs from the ITO electrode with a constant area of 12 mm × 7 mm. All of the kinetic experiments were conducted by employing i*R* compensation to correct the voltage loss caused by the electrolyte resistance between the working and the reference electrodes.

### 4.4 Computational Methods

The ionization, solvation, and ligand exchange energy calculations were carried out with the QCHEM quantum chemistry program package.^58^ The LANL2DZ double-ζ basis set^59^ is used as the effective core potential (ECP) with the PBE functional^60^ for gold as it is a heavier atom. The SCF algorithm used for the geometry optimizations is DIIS with geometric direct minimization and convergence criteria of 10^–6^ au.

The ionization energy is treated as the energy difference between the Au in the neutral state and the Au^3+^ cation, both in a vacuum. For solvation energies, the first hydration shell is added in a straightforward approach, with 6 water molecules around the Au ion/complex. To this, a polarizable continuum model (PCM) is added in the CPCM^61^ level of theory, which is a conductor-like PCM method with a dielectric screening factor: . The geometry, including the first hydration shell, is optimized with the PCM.

The energy difference between the AuCl_4_^–^ complex in solution and the hydrated Au^3+^ cation and 4Cl^–^ hydrated anions, is the energy needed to create the Au complex by exchanging the water with the nonaqueous ligands. This energy is called the ligand exchange energy.^37^ As there are 6 water molecules in the first hydration shell of the Au cation, 4 of these water molecules are exchanged for chlorine atoms to create the AuCl_4_^–^ complex, according to the electrochemical reaction.

#### 4.4.1 Slab Calculations

All calculations for the slab models of Au(111) surfaces with the adsorbed species for the sublimation energy (explained in the Results and Discussion section) were performed by using the periodic plane-wave set code Vienna Ab Initio simulation package (VASP 5.4.4) based on DFT. The exchange-correlation potential was described through the generalized gradient approach (GGA) with the Perdew–Burke–Ernzerhof (PBE) implementation.^62^

Optimal reciprocal space grid sampling was carried out by applying the Monkhorst–Pack^63^ scheme with (9 × 5 × 1) *k*-point grid. The electronic wave functions were expanded in a plane-wave basis set with an energy cutoff of 450 eV. Dipole corrections were applied as the asymmetry of the slabs presents finite-size errors that affect the potential and forces due to the periodic boundary conditions.^64^

Additionally, van der Waals (vdW) interactions were considered with the DFT-D3 method of Grimme with the zero-damping function.^65^

The bulk geometry of gold was calculated with a lattice parameter of 4.17 Å, which compares well with the experimental value of 4.078 Å.^66^ All slab models were composed of 5 gold layers with the bottom 2 layers fixed in place according to the optimized bulk geometry. The top 3 gold layers, as well as all of the adsorbed species were allowed to relax without any further constraints.

The radical species of 2-MBA and 4-MBA as well as the citrate anion were all optimized in a 25 × 25 × 30 asymmetric cell prior to the adsorption studies on the slabs.

The surface slab was modeled with a √3 × 4 unit cell of the Au(111) surface for the 2-MBA and 4-MBA systems. The citrate system was modeled with a larger unit cell of 2√3 × 4. A constant vacuum of ∼29 Å was chosen between two consecutive slabs in the *ẑ* direction as substantial distances were required for the removal of an atom from the slab to the vacuum without it interacting with either slab.

The average binding energy per adsorbed molecule is defined as
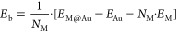
9where *N*_M_ is the number of adsorbed molecules per unit cell, *E*_M@Au_ is the total energy of the adsorbate–surface system, *E*_Au_ is the clean Au slab total energy, and *E*_M_ is the energy of the molecule in a box. For negative binding energies, the adsorption process is exothermic, meaning the adsorbed phase is created during the adsorption process and more negative binding energies imply stronger adsorption between the surface and the molecule.

## Supporting Information Available

UV–visible, ζ-potential, and DLS measurements; TEM and additional LSV measurements; particle charge and Plieth calculations; and additional DFT results such as sublimation energy convergence and atom displacements (PDF)
