# Supplementary material for: The Effect of the Capping Agents of Nanoparticles on Their Redox Potential
Source: J Am Chem Soc. 2024 Jul 3;146(32):22208–19. doi: 10.1021/jacs.4c02524 (PMC11328137; doi:10.1021/jacs.4c02524)
Supplement: Supplementary file 1 — ja4c02524_si_001.pdf [file ja4c02524_si_001.pdf]

The Effect of the Capping Agents of Nanoparticles on Their Redox Potential

Pavel Savchenko▿, Din Zelikovich▿, Hadassah Elgavi Sinai, Roi Baer*, Daniel Mandler*

Fritz Haber Research Center for Molecular Dynamics and Institute of Chemistry, The Hebrew University of Jerusalem, Jerusalem 9190401, Israel

▿These authors contributed equally.

Daniel.mandler@mail.huji.ac.il

Roi.baer@mail.huji.ac.il

Supporting Information

Ligand Type	2-MBA	4-MBA	cit	MPA	MAA
**Peak Potential/mV**	0.920±0.007	0.991±0.007	0.962±0.003	0.94±0.01	0.946±0.001
**Diameter/nm**	10.8±1.2	11.0±0.9	10.5±0.3	10.4±0.5	10.2±0.6
**ζ-potential/mV**	-49.1±2.0	-46.5±2.9	-47.3±1.8	-43.1±1.4	-42.5±1.2

Table S1: The average of the oxidation peak potentials, DLS particle size distribution, and ζ-potential for the different ligands stabilizing the AuNPs.

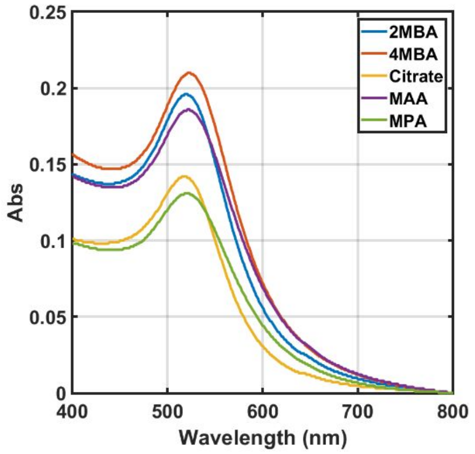

Figure S1: UV−visible spectra of the AuNPs stabilized by citrate and different thiols. λ_{max} for citrate, 2-MBA, 4-MBA, MAA, and MPA are 518, 521, 523, 521, and 520 nm, respectively.

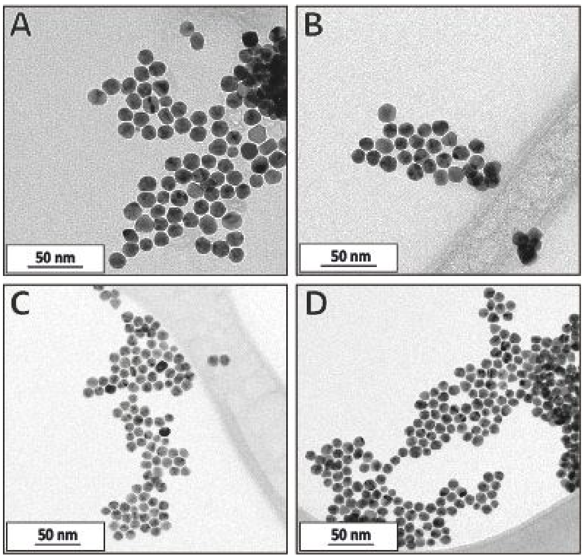

Figure S2: High-resolution TEM images of AuNPs stabilized by (A) cit, (B) MPA, (C) 2-MBA and (D) 4-MBA.

Particles per surface from charge calculation

In order to calculate the amount of AuNPs dissolved from the surface, we look at the total charge, Q_{tot}, by taking the integral of the linear sweep voltammetry (LSV) peak. Q_{tot} – the charge under the LSV curve is proportional to the number of particles that dissolute from the surface through the relation:

$$Q_{tot} = N_{AuNP} \cdot Q_{AuNP} \qquad (1)$$

Where N_{AuNP} is the total number of AuNPs that are immobilized on the surface and Q_{AuNP} is the charge achieved by oxidizing a single particle. By considering the full oxidation of all atoms in the NP to Au^{3+}, the charge Q_{AuNP}, can be expressed as:

$$Q_{AuNP} = N_{atoms} \cdot 3e^- \qquad (2)$$

Where $e^- = 1.6 \cdot 10^{-19}\ C$ is the charge of an electron and N_{atoms} is the number of atoms in a single particle. To calculate N_{atoms}, we start by looking at the unit cell of FCC Au with a lattice constant of 4.078Å.[1] The volume of each unit cell in the lattice is 67.82 Å^3, and there are 4 total atoms per unit cell.

Therefore, we get:

$$N_{atoms} = \frac{V_{AuNP}}{V_{uc}} \cdot 4 \qquad (3)$$

Where V_{AuNP} is the volume of each particle expressed as the volume of a sphere ($V = \frac{4\pi}{3} r^3$) and V_{uc} is the volume of the unit cell.

Assuming the mean radius of a particle across the surface is 50Å, the charge of a single particle derived from equation 2 and equation 3 is:

$$Q_{AuNP} = \left(\frac{\frac{4\pi}{3} \cdot (50\text{Å})^3}{67.82\text{Å}^3} \cdot 4 \right) \cdot 3e^- = 1.48 \cdot 10^{-14} C \qquad (4)$$

The results for the number of particles oxidized per cm2 is given in Table 4.

Ligand	Total oxidation charge (μC)	Mean NP radius (Å)	Q_{AuNP} (μC)	Number of Particles/cm^2
**Citrate**	1.7182	50.97±5.32	$1.57 \cdot 10^{-8}$	$1.29 \cdot 10^{10} \pm 0.13 \cdot 10^{10}$
**2-MBA**	8.5114	47.84±6.37	$1.29 \cdot 10^{-8}$	$7.78 \cdot 10^{10} \pm 1.03 \cdot 10^{10}$
**4-MBA**	3.2442	50.57±8.23	$1.53 \cdot 10^{-8}$	$2.51 \cdot 10^{10} \pm 0.40 \cdot 10^{10}$
**MPA**	3.8060	51.07±4.07	$1.58 \cdot 10^{-8}$	$2.87 \cdot 10^{10} \pm 0.23 \cdot 10^{10}$
**MAA**	7.1612	51.14±4.43	$1.58 \cdot 10^{-8}$	$5.37 \cdot 10^{10} \pm 0.46 \cdot 10^{10}$

Table S2: Comparison of number of particles per surface derived from equation 1 and equation 4. Mean NP radius is taken from size statistics of the SEM images of the different surfaces and total oxidation charge is integrated from Figure 1 curves.

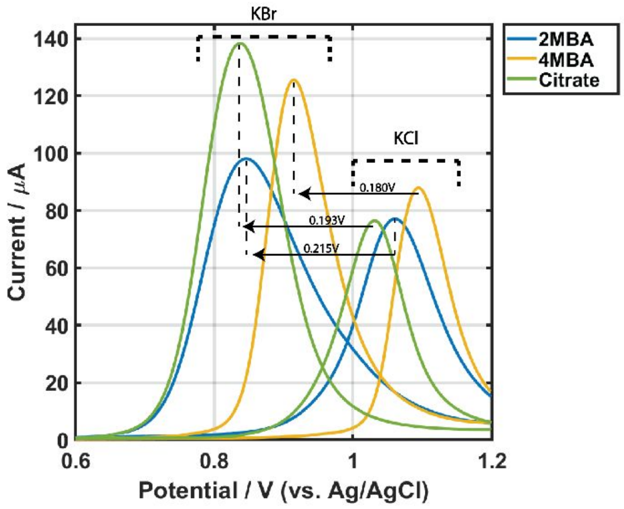

Figure S3: LSV scans of ITO/PEI/AuNPs stabilized by cit/2-MBA/4-MBA in KCl and KBr electrolytes, showing the relative shifts.

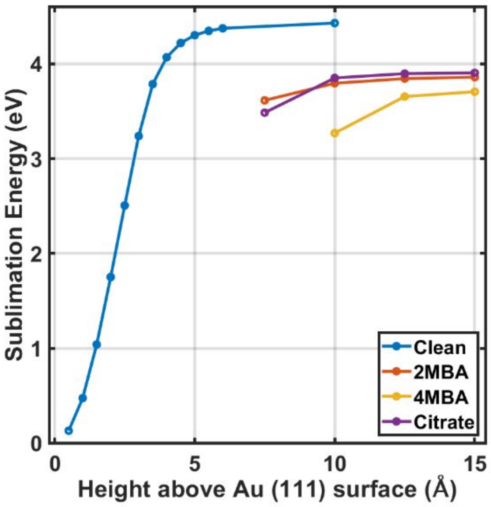

Figure S4: Sublimation energy convergence vs. the height of the detached atom above the gold surface.

The curve for the clean Au (blue) is smooth as the surface slowly adapts to the elevation of the atom, and at around 5Å above the surface, the system energy converges. For the different ligands, the energy converges only when putting the loose atom higher, as there are geometric constraints of the molecules. For example, the 4-MBA molecule (yellow), which “stands” up as it is ordered in a self-assembled monolayer, converges only at larger distances from the slab itself where there is no interaction with the molecular layer.

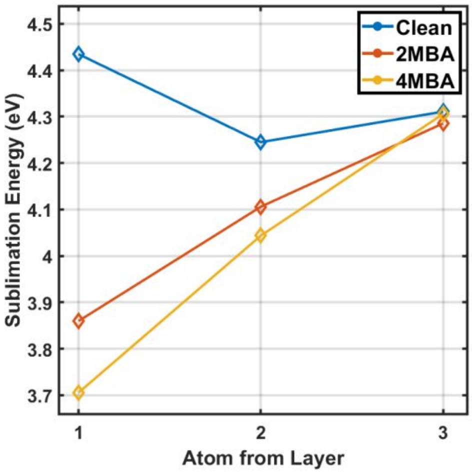

Figure S5: Sublimation energy vs. the layer from which the atom was removed. The weakest bound atom was chosen for each of the layers.

The sublimation energy for the third layer converges into a single value for all the different surfaces, as the effects inside the Au are not influenced by the surface chemistry but rather by the bulk metal.

For the 2-/4-MBA ligands the sublimation energy increases with the depth, which implies that the surface atoms are more likely to detach during the oxidation process, reinforcing the idea that atoms are initially extracted from the surface.

The clean Au on the other hand, shows a different behavior. The lowest sublimation energy is found for the second layer, whereas that of the third layer converges with the bulk. This phenomenon can be explained by the surface reconstruction of Au (111). The surface undergoes a surface reconstruction for the $22 \times \sqrt{3}$ supercell, where an additional atom from the bulk diffuses to the surface.[2 3] This shows that the $\sqrt{3} \times 4$ unit cell is unstable and the Au atom is therefore removed from the second layer in this case.

Plieth equation

Plieth's equation is:

$$V_{\mathrm{NP}} = V_{Bulk} - \frac{2\gamma V_m}{zF} \cdot \frac{1}{r} \qquad (5)^{4}$$

Where V_{NP} and V_{Bulk} are the oxidation potential of the AuNP and bulk gold, respectively. γ is the surface tension, V_M is the molar volume, z, and F, are the number of electrons and Faraday's constant, respectively. Finally, r is the radius of the NP.

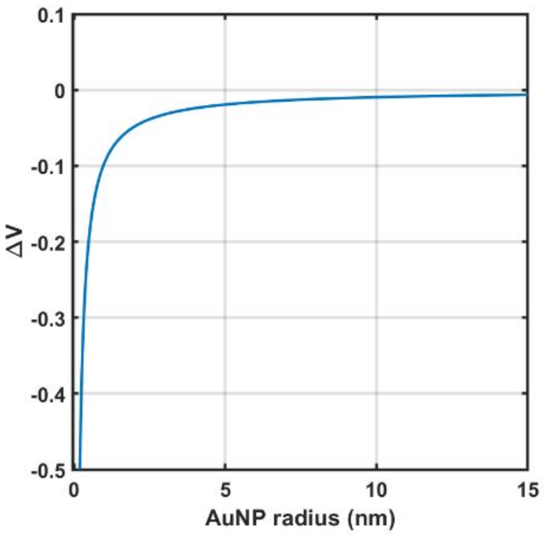

Figure S6: The oxidation potential shift of AuNPs as a function of their radius according to equation 5. For AuNPs with a diameter of 10 nm, the potential shift is −19.33 mV.

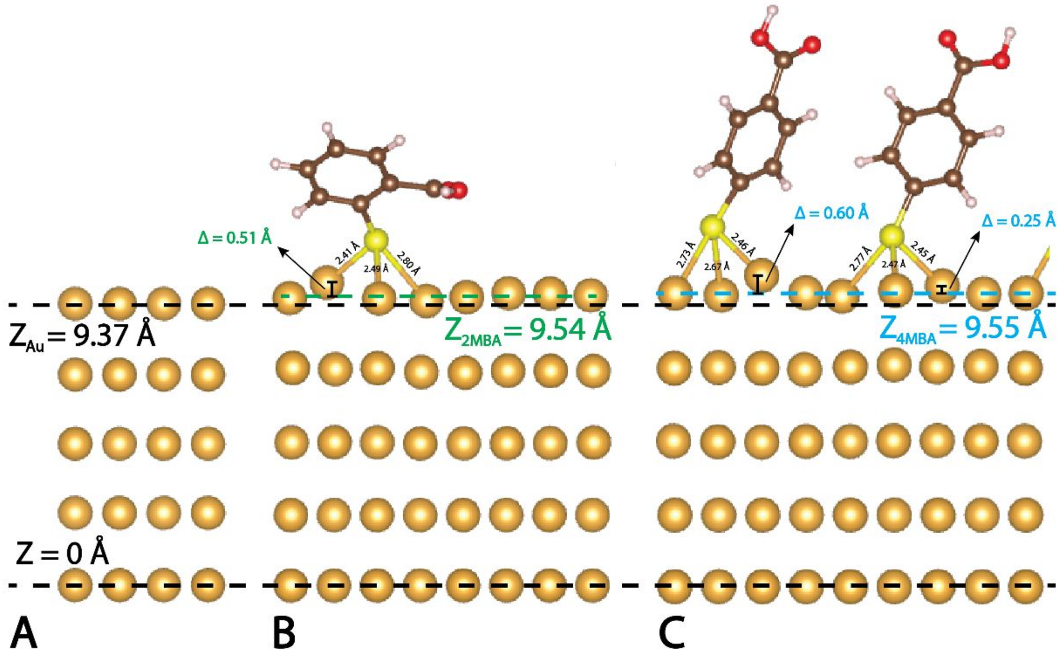

Figure S7: The displacement of the top Au layers of the electrode, by the adsorbed molecules. (A) Is the clean Au surface with the top layer at Z=9.37 Å. (B) Represents the lifting of the gold due to the adsorption of 2-MBA. The average of the lifted Au layer height is Z=9.54 Å, with the highest Au atom 0.51 Å above the average. (C) Shows the lifting of the Au by adsorbed 4-MBA. The average lifted Au layer height is Z=9.55 Å, with two Au atoms lifted 0.60 and 0.25 Å above the average for the FCC and HCP sites, respectively.

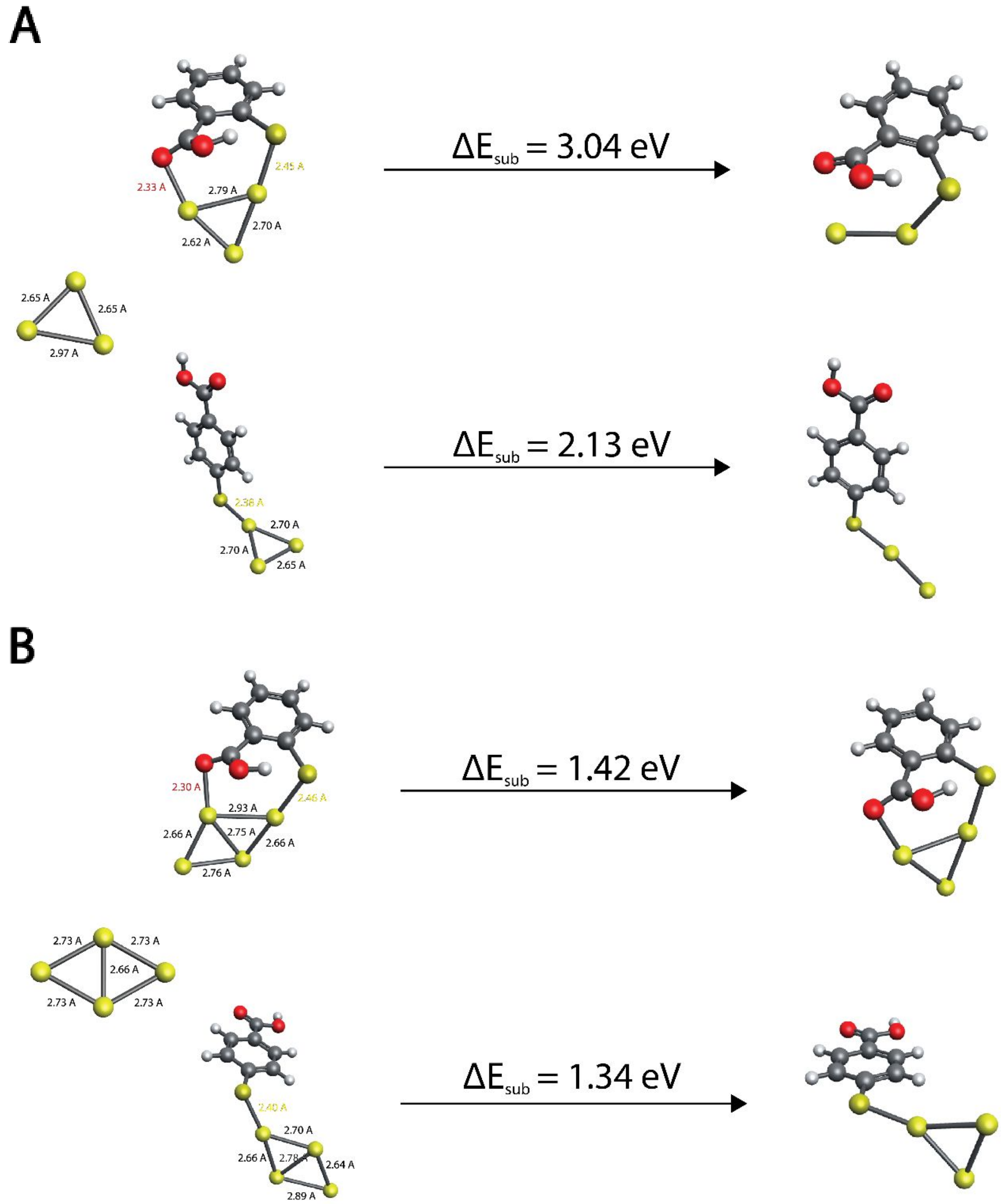

Figure S8: Calculation of sublimation energies and Au-X bond lengths (X: Au, O, S) for small Au clusters of (A) 3 Au atoms and (B) 4 Au atoms, connected to 2-MBA (top) and 4-MBA (bottom).

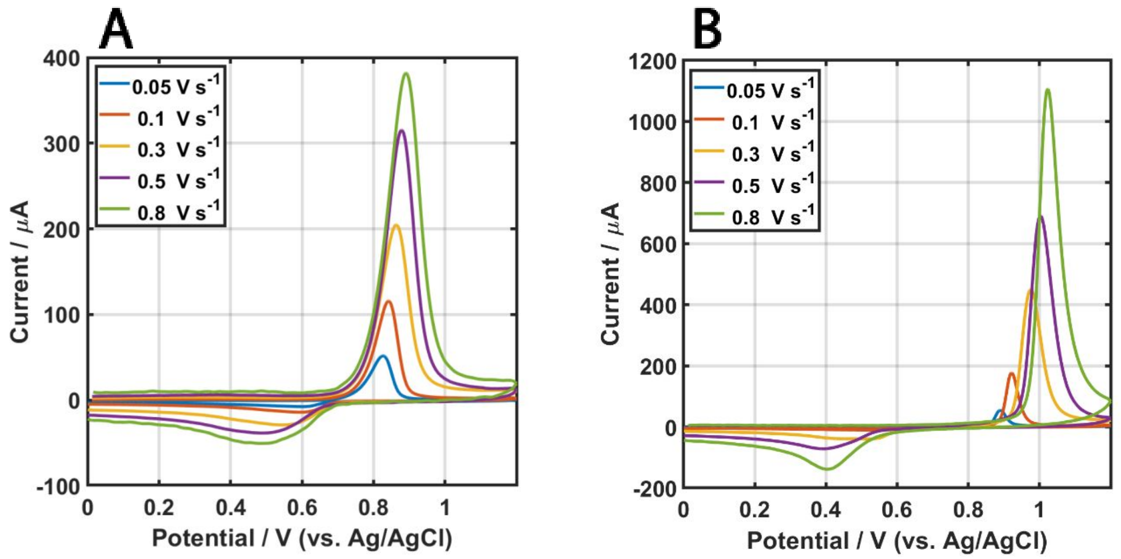

Figure S9: CV acquired with ITO/PEI/AuNP electrodes stabilized by (A) citrate and (B) 4-MBA at different scan rates.

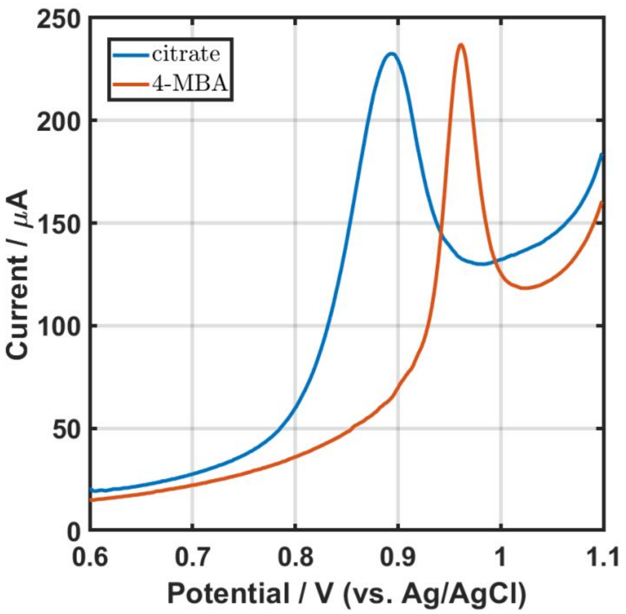

Figure S10: LSV scans of Pt/PEI/AuNPs stabilized by cit/4-MBA in 0.1M HCl solution.

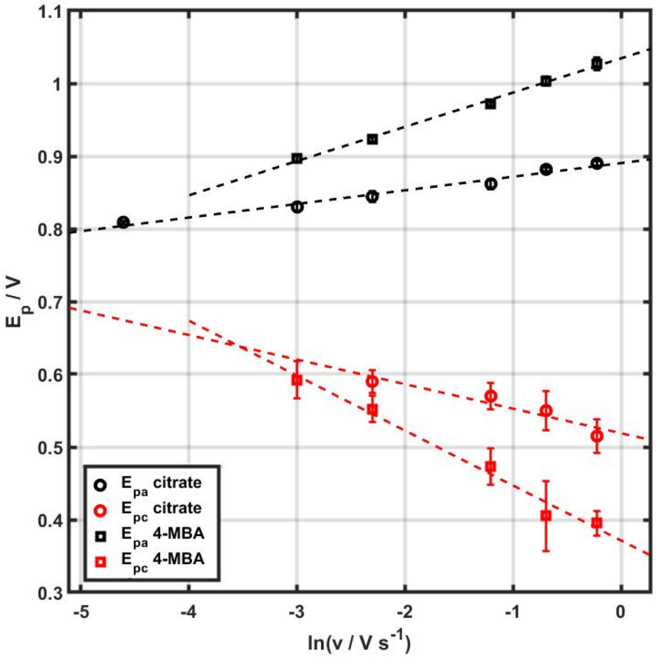

Figure S11: E_p vs. the natural logarithm of the potential scan rate for the data shown in Figure 7 and their linear fits.

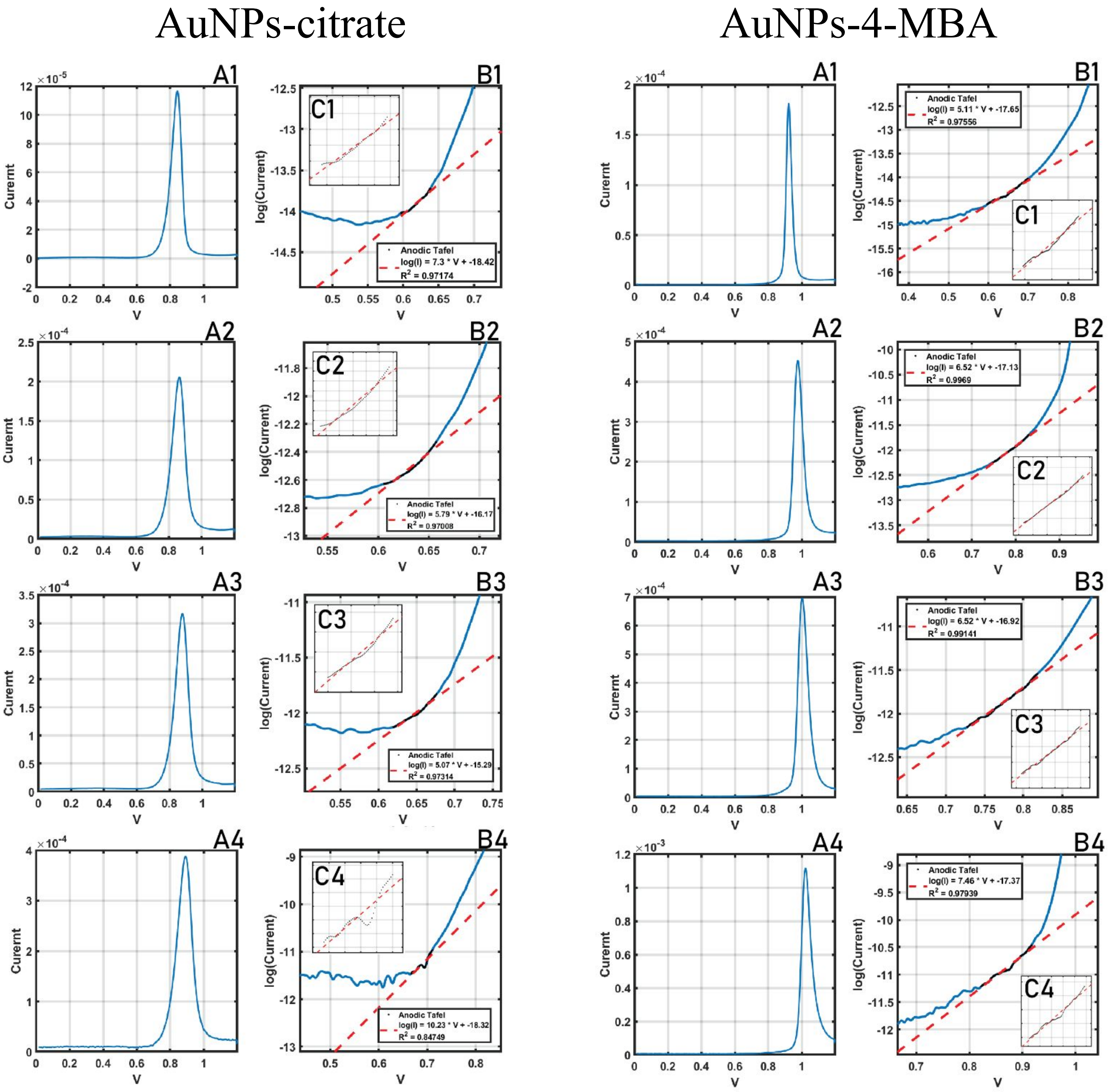

Figure S12: (A) LSV of ITO/PEI/AuNP electrodes stabilized by (left) citrate and (right) 4-MBA scanned at different scan rates (1-0.1 V/s, 2-0.3 V/s, 3-0.5 V/s, 4-0.8 V/s), (B) Tafel slope determined from the horizontal region (kinetic region) in the logarithm of the current from the LSV vs. the potential, (C) magnification of the kinetic linear region.

References

.1 Pearson, W., Lattice spacings and structures of metals and alloys. *Vols. I and II (Pergamon Press, Oxford, 1964, 1967)* **1958**.

.2 Hanke, F.; Björk, J., Structure and local reactivity of the Au(111) surface reconstruction. *Physical Review B* **2013,** *87*.235422 ,(23)

.3 Li, P.; Ding, F., Origin of the herringbone reconstruction of Au(111) surface at the atomic scale. *Science Advances* **2022,** *8* (40), eabq2900.

.4 Plieth, W. J., Electrochemical properties of small clusters of metal atoms and their role in the surface-enhanced Raman scattering. *The Journal of Physical Chemistry* **1982,** *86* (16), 3166-3170.
